# Medical Electricity

**Published:** 1869-04-15

**Authors:** 

**Affiliations:** Academy of Sciences, Paris


### TRANSLATIONS.

### *Medical Electricity.*

*The State of our Knowledge Concerning the Application of Electricity to the Treatment of Disease.*

REPORTED TO THE ACADEMY OF SCIENCES, PARIS, BY M. BECQUEREL.

### I.—Electro-Therapeutics Previous to the Discovery of the Pile.

Whenever an energetic agent is discovered in nature, the physician, who seeks to relieve the sufferings of his patient, tests its action upon diseased organs, in the hope of affecting a cure vainly attempted by medical science. The attempts succeed, observed facts are associated, are co-ordinated, their laws and relations are reduced; there science commences where empiricism ends. The application of electricity to therapeutics is yet in its first phase, although it has already given satisfactory results in certain cases; that they are not very numerous, is attributable, doubtless, to the very complicated effects of this mode of treatment.

The Greeks, more than six centuries before the Christian era, recognized the property, which amber possesses, when rubbed, of attracting light bodies which may be presented to it; credulous of the marvelous, they attributed to every object a soul, to which they assigned miraculous qualities.

From the time of Pliny amber was already sought for its medicinal properties; women and children, in special cases, wore collars of this substance, a custom handed down to our own time, but now almost abandoned. Appian relates that the shock of the torpedo was used for the cure of the gout and of paralysis; a shock which is identical with that of the Leyden-jar.

Vossius adds that in his time it was used for the cure of inveterate headache. To-day electricity is applied to the same diseases.

It appears, according to Thompson, the historian of the animals of western Africa, that from time immemorial, the negro races of central Africa took advantage of the electrical properties of the silurus for the healing of sick children; the children are placed in a bath filled with water, with this fish, which makes its discharges from time to time; the electricity then, perhaps, acts only as an excitant of movements in the muscles, as in gymnastics.

Centuries must be passed before we reach the era of the discovery of the Leyden-jar, 1746; an epoch when the applications of electricity to therapeutics were so greatly extended, that they were then persuaded that the electrical agent was the principle of organic life.

This remarkable experience produced such an effect upon those who first received the shock, that Muschenbrœck wrote to Réaumur that he would not repeat it were all France to be given to him.

The moral effect was such that he was breathless, and that, two days afterward he had scarcely recovered from the emotion and from the disturbance which he had experienced. Winkler asserted, moreover, that the first discharge from the Leyden-jar had occasioned a cramp throughout his entire body, and that his circulation had been so excited thereby that, fearing a high fever, he had resorted to refrigerant remedies. The prejudice against the dangers of experiments with the Leyden-jar having been weakened, its medicinal application began to attract attention. Nollet appears to have been the first who applied the electrical agent to therapeutics; he commenced by investigating the effects which it produced upon liquids during prolonged action; he observed that it accelerated their evaporation, and that this was by so much the more rapid, as the surfaces of the containing vessels were extended.

Boze observed, at the same time, that water electrified escaped from capillary tubes in the form of rays, instead of drop by drop, as otherwise.

These experiments, whose results depend upon the repulsion excited between bodies similarly electrified, were regarded as decisive by every physician engaged at that time in the application of electricity to medicine; but they made no deduction therefrom; they believed, for example, that they might conclude from them, that electricity accelerated the circulation of the blood; but experience has not failed to demonstrate the reverse.

Bertholon and Jalabert, like Nollet, applied electrical discharge to the treatment of paralysis.

Animals were killed by means of strong discharges, in order to determine the disorders which they would determine. In a frog whose chest had been opened, the lungs were inflated, aud were expelled from the body, by the repulsive action of electricity; the heart still continued to pulsate for some minutes.

A strong discharge was passed through the head and the body of another frog; there was a sort of distention of all the limbs; one hour afterward they were restored to their original appearance. This was the first instance of tetanus produced by electricity.

Franklin's theory appeared. It assumed that there existed in every body a certain quantity of electric fluid; if this quantity was increased, these bodies were found to be in a state of plus (positive); if it were diminished, they were in a state of minus (negative) electricity. Physicists and physicians, carried away by this theory, imagined that when the body of man ceased to be in its normal condition, by any disturbance whatsoever of its functions, there was diminution of the electric fluid; in this case it was necessary to administer an additional quantity. This theory, which is now abandoned, is still, however, sustained by some physicians.

In order to apply electricity to the healing art, machines, sufficiently powerful to furnish a continuous current of sparks, more or less strong, Leyden-jars of different sizes, a cushion and excitors of different forms, were then used, which had been indicated as infallible means of cure. With jars shocks were given; with excitors sparks were drawn from different parts of the bodies of patients. Again, electricity was administered in the form of a bath, as is still practiced to-day. It was believed to have been established that electricity was of some utility:

1st.—In contractions which depended upon affections of a nerve.

2d.—In sprains, etc., when the inflammation is passed.

3d.—In indolent tumors.

4th.—In some cases of paralysis.

But it must be stated, these different modes of treatment were not preceded by physiological experiments.

The remark may be made cursorily that these pathological cases are precisely those in which electricity is applied to-day.

Such was the condition of electro-therapeutics, when Volta made his admirable discovery.

**II.—Electro-Physiological and Electro-Therapeutical Investigations since the Discovery of the Pile.**

Galvani having discovered, in 1790, that by arming the muscles and the nerves of a frog, properly prepared, with two different metals, of which one only was oxydizable, as has been recognized subsequently, their simple contact would suffice to produce contractions; this fundamental experiment was the point of departure for the discovery of the pile.

According to Galvani all animals possess specific electricity, which is secreted in the brain, and resides in the nerves, which transmit it to all parts of the body. The common reservoirs are the muscles, of which each fibre should be considered as possessing two surfaces, upon each of which is found one of the two electricities; hence, he compared the muscles to a small Leyden-jar, of which the nerves are the conductors. He believed that the electric fluid was drawn from the interior of the muscles into the nerves, and from these to the exterior surface of the muscles, from whence there resulted an electrical discharge to which corresponded a muscular contraction.

I only mention this theory because it served as a point of departure for all physicians who were, at that time, engaged in galvanic investigation. When it was announced, a struggle commenced between Galvani and Volta. The latter proved that the electricity produced by the contact of the two metals, that is to say by the oxydation of the zinc, was the cause of the contraction. At one time Galvani was considered victor, when he proved, aided by his nephew, Aldini, that the metallic arc was not necessary to excite contractions, since they were obtained in a frog newly prepared, by placing the crural muscles in contact with the lumbar nerves. Volta replied that this was only a generalization of his principle, according to which all bodies sufficiently good conductors established themselves always, by their mutual contact in two contrary electrical conditions; but Volta was deceived. Galvani had just discovered, conjointly with Aldini, the proper current of the frog, of which Nobili, Marianini, Matteucci and DuBois-Reymond had made a most thorough study. This discovery is doubtless one of the most important which has been made in electro-physiology, for if one day we should arrive at the discovery of the intervention of electricity in the phenomena of life, this discovery would have been the point of departure for investigations which would have been made in this direction. The discovery of the pile startled the School of Medicine of Paris, which nominated a commission to repeat all the experiments made upon galvanism since 1790.

This commission determined that the electricity of the pile penetrates the nervous and muscular organs more profoundly than that from the ordinary electrical machines, and that it provoked lively contractions, strong pricking and burning sensations in localities whose diseased conditions rendered them otherwise insensible to sparks, or to ordinary shocks.

The National Institute, aroused by the general movement which the effects of galvanism had initiated, nominated, in 1799, a commission composed of Coulomb, Sabattier, Pelletan, Charles, Fourcroy, Vauquelin, Guyton and Hallé, to examine and verify the phenomena of galvanism. This commission composed of the most eminent men of the epoch, established a distinction between the electric and the galvanic fluid; it assumed to perceive in the animal organization, a principle in which resided the essence of the mutual relations of the nervous and the muscular systems. The animal arc may be formed of nerves and muscles in mutual contact, as Galvani had discovered. This arc is not interrupted by the section of a nerve or its ligature, provided that the part remain in mutual contiguity during muscular action. It is not so, however, in the case of the living animal, since it suffices to cut a nerve in an animal, or to enclose it in a ligature, in order to deprive it of the faculty of communicating motion to the muscle to which it is distributed. It recognized the fact that galvanic influence appeared to be excited by exercise, and to be recuperated by repose; and here was, for the first time, indicated the fact resulting from the action produced upon a nerve by a continuous current.

The commission advises, very judiciously, in order to secure accuracy in the experiments and in their appreciation, that the state of health of the animal be previously assured, and also the manner in which it has been preserved and nourished up to the moment of the experiment; experimenters have not always had due regard to this wise recommendation.

The commission of the Institute then investigated the application of electricity as a physiological agent, in a scientific spirit.

No estimate can be formed of all the experiments which were made at this epoch, and which led to results too thoroughly forgotten at this day. We quote two examples only:

Wilson Philips, having cut the nerves of the eighth pair of a rabbit, discovered that by reuniting the two extremities by means of a metallic wire, and by transmitting through them a current, digestion and respiration, which had hitherto been difficult, became easy as soon as the pile was put in operation.

Dr. Andrew Ure experimented upon the body of a criminal immediately after execution, with a pile composed of a large number of elements strongly charged. One of the poles being put in communication with the spinal cord, the other with the sciatic nerve, all the muscles of the body contracted simultaneously, with convulsive movements. Ure succeeded in imitating, up to a certain point, the play of the lungs, by transmitting a current from the spinal cord to the ulnar nerve, he caused the fingers to move with agility; by transmitting a discharge from one ear to the other, and moistening them with salt water, the muscles of the countenance assumed horrible contractions; the action of the eyelids was very marked. This is the first example of the mode of localized electrization at the present day, a mode which was formulated in 1834, in these terms: (Ann. de Chem. et de Phys., 2d série, t. LXVI, p. 27,) by M. Masson, pupil and friend of our celebrated confrère, Savart:

"The property in the induced current of affecting only the points touched, permits the submission to its action of any portion whatever of the body. Thus, by placing two metallic plates upon the extremities of a finger, after having placed them in the current, this latter will only traverse the finger. The importance of this discovery will be readily recognized by those who are engaged in the application of electricity to medicine."

Let us proceed to the application of Voltaic electricity to therapeutics.

Plaff applies it to paralysis of the optic nerve, as Magendie has since done succcssfully, when the paralysis is incomplete.

It has been employed advantageonsly in paralysis of the extremities, in feebleness of vision, and in amaurosis due solely to defective irritability of the optic nerve; in deafness dependent upon diminished nerve-power; in coryza, and in aphonia; in paralysis of the sphincter ani, and in that of the bladder.

Many other applications have been made, and have demonstrated that actual practitioners traverse the same circle as their predecessors.

Have they obtained more or less success than the latter? Statistical records fail to answer the query. Doctor Fabre Palaprat, subsequently, making use of interrupted voltaic currents, at more or less closely approximated intervals, obtained decided effects in cases of atony or feebleness in the functioning of organs, provided that no lesion or inflammation existed, as well as in some of lymphatic engorgement.

Let us delay a moment before making an exposition of the results obtained by eminent physiologists, who have furnished the data by means of which electricity is more methodically applied to therapeutics to-day than formerly, in order to recall facts which it is necessary to take into consideration, when it is desired to compare physiological effects produced by electrical action to those resulting from mechanical, physical, chemical or vital actions.

Animals have excitable parts, sensitive parts, and parts deprived of these faculties. Haller, who is continually encountered in the paths which lead to physiological experiment, dissected the parts and applied the scalpel, acids, or other chemical agents, in order to detect the special properties of each one of them. He thus examined parts which were agitated, and those which experience sensations of pain. By irritating a nerve or one of its ramifications in a muscle, there resulted an abrupt and rapid movement; when a nerve corresponding to a muscle was too strongly irritated, and for too long a time, it ceased to contract. The nerve being cut, if it was irritated below the section, the animal experienced no sensation; but the muscle contracted immediately. If the irritation was effected above the effect was inverse. Electricity produced, nearly always, similar results.

Ligation of a nerve arrests the action of the current, as does that of other stimulants; provided it be sufficiently firm. In this case, after detaching the ligature, it is no longer possible to excite contraction by irritating the nerve above the ligature.

M. Matteucci has recognized the fact that poisons do not all act in the same manner, and that, when the animal is killed by electric discharges, the excitability of the nerve by the current is destroyed. This observation ought to be taken into consideration in this regard, that it shows the danger of exciting the nerves too strongly.

Twenty-five years ago (in 1841), in a memoir crowned by this Academy, our associate, M. Longet, demonstrated experimentally the independence of muscular irritability, and of excitability of the motor nerves. This important fact has been confirmed subsequently by our associate, M. Claude Bernard, by the aid of curare; he has, indeed, perceived that the muscles may remain contractile, even when the motor nerves are no longer excitable. The electric current appears to have been the only one of all the excitants tried, applied to muscles, which could induce their contraction without the intervention of nerve fibres. This fact is very remarkable in this regard, that it seems to establish an analogy between the mode of action of electric currents, and that of nerves in producing muscular contraction.

It has been already seen that a nerve too strongly irritated lost the faculty of exciting contraction in the corresponding muscle, and recovered it by rest. The same is likewise true when the current generated by a certain number of pairs has circulated for a certain time between the muscle and the nerve; the muscle no longer contracts at the opening or the close of the circuit; but if the direction of the current be changed the contractions manifest themselves anew. By reversing, a certain number of times, the direction of the current, the excitability of the muscles of the frog may be arrested or revived at will; it is this which constitutes what is called the phenomenon of voltaic alternatives; but if the organs of a frog, traversed during a certain period by a current of determinate intensity, lose the contractile faculty they have, nevertheless, the power of contracting under the influence of a more energetic current.

The muscles of a frog, which have lost their contractile faculty by the prolonged passage of a current, recover it by repose; the same is true of a living animal; but it is necessary to take into consideration the will of the animal, which might modify the effects of currents up to the point of neutralizing them entirely, especially if the currents have no great intensity, and the animal has a high degree of vitality.

Marianini and other physicians have observed that if the current is directed into the nerve alone, following the directions of the nervous ramifications, that is to say, from the head to the extremities, there is contraction upon closing the circuit and no effect upon interrupting it. If the current traverses in the reverse direction, there is no contraction upon closing the circuit, it is manifested, however, upon interrupting it. There is absence of contraction when the nerve is affected normally longitudinally, as M. Matteucci has demonstrated.

Marianini discovered, moreover, that the current, according to its direction, produced either contractions or painful affections in the frog, as well as in other animals; when the current is direct, going from the head to the extremities, there follow vigorous contractions of the posterior extremities, from the closure; upon opening the circuit the contraction is weaker, the dorsal column bends upon itself sustains a violent shock, and, it sometimes happens that the animal cries out. With the reverse currents opposite effects occur.

It seemed, then, that the nerve was organized in such a manner as to propagate certain movements in the direction of its ramifications, movements which are only transmitted with difficulty in the opposite direction, and from which results the sensation of pain.

Nobili succeeded in giving tetanus to a prepared frog by interrupting and re-establishing the current rapidly. This effect is due, probably, to change in the condition of the nerve, which passes rapidly from the natural to an artificial state, and reciprocally. It may be asked if tetanus, natural to men and animals, would not originate from similar modifications consequent upon acute pain. If it were so, it could be arrested by utilizing this fact discovered by Nobili, that frogs having tetanus persist in this condition under the influence of a current of a certain intensity, and are frequently completely distended under the action of a current directed in the reverse direction. Experiments conducted in this direction have already given satisfactory results.

The existence of the proper current of animals, as has been previously recognized, was indicated and put in evidence for the first time by Galvani; it was investigated successively by Nobili, Matteucci, and DuBois-Raymond; each had his part in the analysis of this important discovery, by the aid of which it was determined that nerves and muscles are electrometers; that is to say, that they are constituted in such a manner as to afford currents when they form closed circuits; these electro-motors probably sustain a rôle still less understood in the phenomena of life, as their organization would suggest.

Nobili perceived that the contraction produced by contact of the crural muscle and the lumbar nerve was due to an electric current, whose existence he had established, a current extending from the paws to the head; the nerve is, therefore, negative.

M. Matteucci established this fact with the living frog; he showed that the proper current of the frog was not weakened by allowing it to circulate through the animal pile, which will be discussed hereafter, whence he concluded that the extremities of the animal do not paralyze in an appreciable manner; an observation which has its significance, for, were it otherwise, it could not be conceived how the muscles and the nerves could intervene amidst vital phenomena as electro-motors if they did always intervene, since polarization would produce an inverse current which would weaken their action at every instant.

M. Matteucci next discovered that muscles are electro motors, since a current could be obtained by placing in communication the interior of a muscular mass with its surface. Nobili obtained a stronger current by forming a coronal pile of cups with pairs composed each of a thigh and the corresponding nerve.

M. Matteucci having placed the nerve of a frog prepared after the mode of Galvani, that is to say, the lumbar nerve still connected with a mass of crural muscle, upon the muscle of another frog, he perceived the former contract at the instant when the last muscles were caused to contract mechanically; it might be inferred from this that the contraction of the muscle produced an electric current, reacting upon the galvanoscopic frog. M. DuBois-Reymond having studied this effect deduces therefrom the following consequences:

The transverse section of a muscle is negative, and the longitudinal positive; nerves having no natural transverse section, they must be cut in order to have a current. These laws appertain to the most intimate constitutive elements of muscles and nerves. The electro-motor power ceases after death, when the muscles and nerves have lost their irritability.

At the moment of contraction a sudden and great diminution in the muscular current and in the nerve is detected, when it transmits a motion or a sensation.

A difference exists between muscle and nerve in their electrical relation; when a nerve is traversed throughout a portion of its length by a continuous current, according to its direction, it increases or diminishes the influence of the proper current. This condition cannot occur in muscle.

Nerves of motion and of sensation act similarly.

The investigations of Jean Müller and of M. Longet into the employment of electricity, for the distinguishing of nerves of motion from nerves of sensation, should be mentioned here, in consequence of their importance in electro-therapeutics. M. Longet has likewise made profound investigations upon nerves of sensation, which possess great interest, and which we recommend to the attention of physiologists.

Neither should reference be omitted to the curious experiments of M. Helmholtz, relative to the duration of the phenomena of muscular contraction, and of the transmission of nervous excitation. By means of ingenious processes and apparatus he succeeded in perceiving that the rapidity of the propagation of nervous excitation in the sciatic nerve is about thirty metres per second. Cooling the nerve greatly diminishes this transmission.

### EDITORIAL.

In compliance with the wishes of several distinguished members of the profession we crowd out of our columns a large amount of valuable matter in order to give place to the correspondence of Drs. Baldwin and Nott in relation to the next meeting of the Am. Medical Association, feeling that the large majority of our readers will unite with us in a cordial endorsement of the sentiments expressed therein.

